# Obituary for Prof. Dr. Alexander Gaskov

**DOI:** 10.3390/s21092913

**Published:** 2021-04-21

**Authors:** Marina N. Rumyantseva, Roman B. Vasiliev

**Affiliations:** Laboratory of Chemistry and Physics of Semiconductor and Sensor Materials, Chemistry Department, Moscow State University, 119991 Moscow, Russia; romvas@inorg.chem.msu.ru


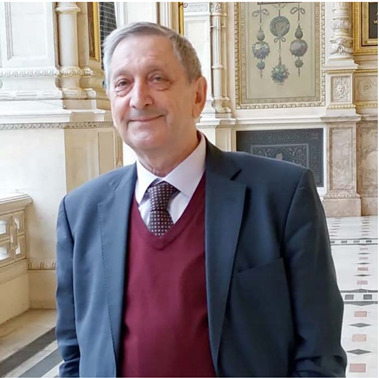


Alexander Gaskov, Prof., Dr. Sci., 1944–2021.

Professor Alexander Gaskov, our dear colleague, friend and teacher, passed away on 18 January 2021, from COVID-19. He was a brilliant scientist in the area of chemistry of semiconductor materials for physical and chemical sensors. He spent his entire research career, which began in 1966, in the Division of Inorganic Chemistry of the Department of Chemistry, Moscow State University. During all his career, he kept generating new ideas, seeking new directions in research and the application of results in practice. He generously shared his knowledge with students and staff, resulting in the emergence of dozens of talented new researchers. The publications produced by the laboratory achieved international recognition. 

Alexander Gaskov is an Eastern Siberia-born scientist. While at school, he showed an aptitude for physics and chemistry. He graduated from high school with a gold medal. In 1961, as the winner of the School Chemistry Olympiad, he was awarded a grant for a trip to Moscow, which he used to enter the Moscow State University (MSU). The entire life of Prof. Alexander Gaskov was inextricably linked to the MSU, at which he has passed all the ranks from a Junior Researcher to the Chief Researcher and Professor of the Moscow State University. In 1966, he was awarded a Ph.D. in Chemistry. The beginning of his research activity was devoted to the development of materials for IR photodetectors and emitters based on single crystals and epitaxial heterostructures of lead and tin chalcogenides. In 1988, he successfully defended the thesis “Directed synthesis of three-component solid solutions of lead and tin chalcogenides for optoelectronics” and was awarded the Advanced Doctoral Degree in Chemistry, which is the highest academic award of the USSR/Russia. In 1993, he received the title of Professor in Inorganic Chemistry. In 2008, Prof. Gaskov was appointed to lead the newly established Laboratory of Chemistry and Physics of Semiconductor and Sensor Materials.

In the 1990s, Prof. Gaskov began exploring a new area of research of the synthesis of nanocrystalline semiconductors with high gas sensitivity. Comprehensive experimental studies of the wide-band semiconductor metal oxides allowed determining an innovative strategy for a direct synthesis of nanocrystalline materials for the chemical gas sensors [1]. For a large number of nanocomposites based on nanocrystalline semiconductor oxides, it was shown that their phase composition, the chemical composition of the surface of the nanocrystals, the nature of the mutual distribution of components, as well as the real structure are determined by the conditions of the synthesis. In due turn, these parameters determine the reactivity of materials and the mechanism of their interaction with the gas phase, the concentration and mobility of charge carriers, and, consequently, the sensor characteristics: sensitivity and selectivity. The revealed correlation between the catalytic activity and the sensor properties of nanocrystalline oxide materials allowed determining the criteria for choosing modifiers capable of increasing the selectivity of semiconductor gas sensors depending on the chemical properties of the target gas.

Later on, the focus of research in the laboratory was placed on active centers located on the surface of nanocrystalline semiconductor oxides [2,3,4]. This led to the development of new in situ and operando techniques for spectral studies of the reactivity of sensor materials in the interaction with gas phase. The trends in active site concentration and sensitivity to various analyte gases were compared across different nanocrystalline metal oxides by using the metal-oxygen bond energy as a descriptor parameter. It was proved that clusters and nanoparticles of noble metals (or noble metal oxides) with specific catalytic activity in the oxidation of certain gas molecules can significantly affect the sensing mechanism.

Research of Alexander Gaskov’s lab in the field of nanocrystalline semiconductors, in addition to oxide materials, involved the binary compounds based on chalcogenides, the so-called quantum dots [5]. These materials are of interest as emitters and photodetectors in the specified spectral regions. Prof. Gaskov initiated the use of quantum dots to promote the photoactivation of gas sensors, which made it possible to reduce the operating temperature to room temperature. Further research was carried-out on the atomically thin colloidal semiconductors complementing the two-dimensional semiconductor systems. In collaboration with other colleagues, a number of new effects were discovered [6].

In the past decade, Prof. Gaskov’s laboratory has been exploring the issues of reliability, stability and power-consumption of semiconductor gas sensors, which are crucial for the successful functioning of sensors in real operational conditions. These areas include the development of synthesis methods that allowed producing the highly dispersed materials in high-temperature processing conditions, as well as the creation of sensitized and hybrid materials with photo-and gas sensitivity for the light-activated gas sensors [7]. The use of the dynamic temperature measurement technique in combination with data processing algorithms has further improved the accuracy and reproducibility of the results of the quantification of target gases in complex gas mixtures which meet environmental and technological requirements.

Although Prof. Gaskov was a strong proponent of fundamental science, he always sought ways of practical implementation of the experimental results. The highly sensitive selective semiconductor sensors created in his lab are the key elements of the Foxy-lab gas analyzer specially designed for detecting highly toxic organophosphorus substances as well as for automatic continuous determination of rocket fuel components. These gas analyzers were installed at Baikonur and Vostochny cosmodromes in 2016 and remain operational.

Alexander Gaskov was a genuine scientist, passionate about his work, with a vision of the global picture, but very strict regarding the accuracy of details. In this vein, he taught his graduate students and this he demanded from colleagues. His enthusiasm and passion for results influenced the people around him, making them achieve and accomplish. As a professor of the MSU, he trained dozens of graduates and post-graduates who opted for specialization in inorganic chemistry, solid state chemistry, and materials science. In recent years, he lectured on the fundamentals of the chemistry of semiconductors, principles of inorganic synthesis, and chemistry of new materials. He was also the organizer and head of the interfaculty course “Functional materials of the XXI century”. Under his supervision, 23 Ph.D. graduates successfully completed their studies. He provided guidance for the two dissertations of the Advanced Doctoral Degree in Chemistry.

Alexander Gaskov was a member of the Academic Council of the Chemistry Department and a member of the Dissertation Council for Chemical Sciences of Moscow State University. In 2009, he was awarded the title of the “Distinguished Researcher of Moscow University”, and 2020, the title of the “Honorary Worker of Science and High Technologies of the Russian Federation” from the Ministry of Science and Higher Education of Russia.

Above all, Alexander Gaskov loved life, his work, his laboratory, and his family. In the “sensor community”, he will be remembered as a genuine scientist who made an important contribution to the understanding of the chemical foundations of the nature of semiconductor materials. By his colleagues, he will be remembered as a leader, teacher and a dear friend. With sadness for our common loss, but with vivid memories and gratitude for Alexander’s precious time lived with us, we will continue our work, bringing his ideas and plans further.

## Data Availability

Not applicable.
